# Supplementary figures and images for: Evaluation of RNA extraction and rRNA depletion protocols for RNA-Seq in eleven edible seaweed species from brown, red, and green algae
Source: PLoS One. 2026 Jan 2;21(1):e0339896. doi: 10.1371/journal.pone.0339896 (PMC12758695; doi:10.1371/journal.pone.0339896)

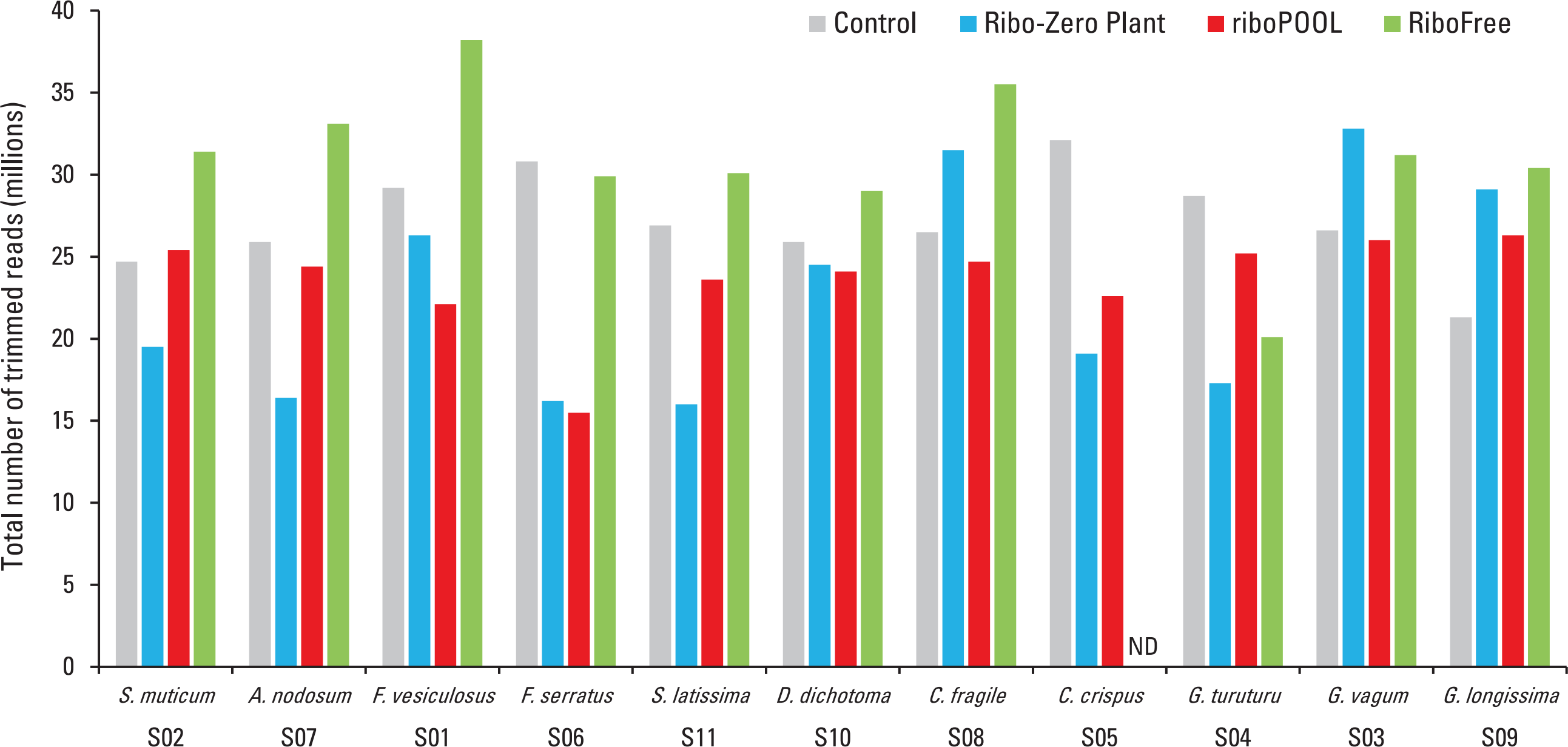

Supplement: S1 Fig — Bar plots represent the total number of trimmed reads for each sample across four ribodepletion protocols: Control (no depletion, gray), Ribo-Zero Plant (blue), riboPOOL (red), and RiboFree (green). Each set of bars represents a distinct seaweed species, identified by its Latin name and sample number (S01–S11; see Table 1), encompassing brown, red, and green algal groups. Sequencing library preparation from Chondrus crispus (S05) total RNA was unsuccessful for unknown reasons and is marked as “ND” (not determined). (PDF) [file pone.0339896.s001.pdf]
