# Supplementary material for: Evaluation of RNA extraction and rRNA depletion protocols for RNA-Seq in eleven edible seaweed species from brown, red, and green algae
Source: PLoS One. 2026 Jan 2;21(1):e0339896. doi: 10.1371/journal.pone.0339896 (PMC12758695; doi:10.1371/journal.pone.0339896)
Supplement: Attachment — Submitted filename: Reviewer Comments_PLOSONE.pdf [file pone.0339896.s008.pdf]

Reviewer Comments

Overall, the manuscript addresses an important methodological framework in algal transcriptomics by systematically evaluating RNA extraction and rRNA depletion protocols across a diverse set of seaweed species. The manuscript is generally well structured and the experimental design appears rigorous. However, there are areas where clarifications are needed before the manuscript can be accepted.

Introduction

Pg. 2, Line No.30-32: The authors stated that "advancing our understanding of seaweed biology, including its response to environmental stressors and pathogens, is essential for enhancing cultivation practices." Please clarify which stressors (temperature, salinity, pollutants) have already been studied in seaweeds, and which remain underexplored?

Pg. 2, Line No.39-44: The authors have mentioned **"**RNA-seq limitations in capturing non-polyadenylated RNAs. Since many algal viruses and prokaryotic RNAs lack polyA tails, could the authors provide evidence from prior algal/seaweed transcriptome studies where viral transcripts were missed due to polyA-based methods?

Pg. 2, Line No.45–61: Have there been any comparative evaluations of these methods specifically in seaweeds before? If not, please make clear that this study is addressing the first comparison report.

Methods

Pg. 11, Line No. **245–251**: The authors mentioned flash-freezing in liquid nitrogen and storage at -80 °C. Since long-term frozen storage can sometimes affect RNA quality in high-polysaccharide tissues, was RNA integrity assessed immediately after storage to ensure no degradation during sample handling?

In CTAB methods, do authors measured the carryover of polysaccharides or polyphenols (e.g., by A230 contamination ratios or carbohydrate assays) to confirm the effectiveness of CTAB-based removal?

In LogSpin method, any RNase inhibitor was added during extraction, since guanidine-based buffers can still leave trace RNase activity in seaweed tissues?

Pg. 15, Line No.350–353, the cutoff of 90% coverage relative to the highest coverage contig was used. How was this threshold determined?

Results and Discussion

Page 5, Line No.108–110: CTAB-based methods generally yielded higher RNA quantities but did not consistently align with acceptable RNA integrity. What underlying molecular or biochemical factors could cause this discrepancy? Please discuss.

Page 5, Line No 111–113: Spin-column methods performed better in red seaweeds. What specific traits of red seaweeds influenced their compatibility with spin-column methods compared to CTAB-based ones?

Page 6, Line No 135-141: Methods involving phenol/chaotropic salts showed suboptimal purity in brown seaweeds. how was this validated? as residual contaminants directly affect downstream ribodepletion or sequencing efficiency.

Overall, the authors need to provide a more detailed discussion of the results obtained.
