# Supplementary material for: Evaluation of RNA extraction and rRNA depletion protocols for RNA-Seq in eleven edible seaweed species from brown, red, and green algae
Source: PLoS One. 2026 Jan 2;21(1):e0339896. doi: 10.1371/journal.pone.0339896 (PMC12758695; doi:10.1371/journal.pone.0339896)
Supplement: Attachment — Submitted filename: Comments RNAseq.docx [file pone.0339896.s009.docx]

**Reviewer comments**

**Abstract**

The genus names of Seaweeds has to be mentioned for more clarity along with the phylum name. (Line nos 17 & 18)

**Introduction**

In Line no. 31 Include as “Environmental Stress factors….”

Line nos 78 – 84 can be removed from Introduction part and may be included in Results and discussion column.

**Results and Discussion**

Nano drop quantification may be included in the methodology for the justification of purity of RNA.

Line no 144 Title has to be precise and meaningful.

Kindly justify the novelty of the work.

Fig 1 & 2 Standard errors may be provided in the figures.
