# Supplementary material for: Evaluation of RNA extraction and rRNA depletion protocols for RNA-Seq in eleven edible seaweed species from brown, red, and green algae
Source: PLoS One. 2026 Jan 2;21(1):e0339896. doi: 10.1371/journal.pone.0339896 (PMC12758695; doi:10.1371/journal.pone.0339896)
Supplement: Attachment — Submitted filename: Response to reviewers.docx [file pone.0339896.s010.docx]

**Reviewer #1**

*The manuscript “Evaluation of RNA extraction and rRNA depletion protocols for RNA-Seq in eleven edible seaweed species from brown, red, and green algae” presents a well-designed comparative evaluation of RNA extraction and rRNA depletion protocols across a taxonomically diverse set of edible seaweed species. The study addresses important technical aspects in algal transcriptomics and provides practical recommendations for future research. However, I have some suggestions and clarification details that authors have to include in the manuscript.*

We sincerely appreciate the reviewer’s time and effort in carefully evaluating our manuscript, and for providing valuable suggestions and corrections that have greatly enhanced its quality.

***1. Is it clear why these specific 11 seaweed species were selected, and are they commercially significant varieties?***

Since using fresh material is essential to minimize effects on RNA quality, we made an effort to obtain locally harvested seaweed in the Netherlands, which is limited due to permit requirements. The species we selected include all those currently harvested by the permit holder (WildWier). These species are mainly collected for food use in the Netherlands, but many are also of global or regional importance outside of the Netherlands (see the FAO report, [The Global Status of Seaweed Production, Trade and Utilization](https://backend.orbit.dtu.dk/ws/portalfiles/portal/163078059/FAO_report_Global_seaweed_2018.pdf)). To clarify our sampling rationale, we have added a brief explanation in the Results section and included this report as a reference (L93–98).

***2. Collection area: Latitude and longitude should be included in the manuscript***

Agreed. We have added site coordinates to the ‘Sample collection and preparation’ section in the Materials and Methods (L303-306). Saccharina latissima was obtained from NIOZ (research facility supply, not a field collection), so no field coordinates apply for that entry.

***3. On what criteria did the authors choose the protocols (RNA isolation) used in this study? The choice of specific CTAB and spin-column protocol variants should be justified, as numerous alternatives exist in the literature.***

We agree that the manuscript would benefit from making our selection rationale more explicit. Indeed many protocols exist, but these have by far mostly been developed for non-algae. At the start of this study, which was not originally designed to tackle RNA isolation issues, we have scanned literature for protocols specifically addressing RNA isolation from macro-algae, including one protocol that is aimed at isolating RNA from strawberry, which is plagued by high polysaccharide/polyphenol levels. The other class of RNA isolation protocols, i.e. based on chaotropic-salt lysis and silica spin columns, is used extensively for many species and tissue types. Among these, the RNeasy PowerPlant kit was specifically designed for plant samples with high polysaccharide and polyphenol content. We included further information on the selection of RNA isolation methods for this study (L315-320).

***4. Include the relevant environmental parameters of the sample collection area.***

Because this study focused on optimizing RNA isolation and ribodepletion protocols rather than testing biological hypotheses, we unfortunately did not collect environmental parameters. The only contextual information we can still obtain at this time is the classification of collection sites as tidal marine or saline lagoon, including the exact location coordinates. We have added this to the Materials and Methods (L303-308).

***5. The use of the 28S/18S ratio over RINe values is appropriate; however, a brief explanation of how plastid-derived rRNA interferes with RINe scoring in algae would improve accessibility for readers outside the field.***

We agree and have added an improved explanation (L128-133) to clarify why RINe is unreliable in seaweeds.

***6. The limitations of this method (constructing an rRNA), particularly with incomplete rRNA assemblies, should be acknowledged in the discussion.***

We appreciate the reviewer’s point. In the original manuscript we discussed only one limitation of our rRNA proxy, i.e. false positives arising when some non-rRNA reads map to rRNA references. We agree that the complementary issue, incomplete coverage of the full set of nuclear, plastid, and mitochondrial rRNAs leading to false negatives, was not explicitly addressed. We have now added text in the discussion section (L213-221) noting that such incompleteness could cause underestimation of absolute rRNA fractions and that these values should be interpreted as lower bounds. Importantly, the same proxy and reference set were applied uniformly to all samples, including the no-depletion controls, so any residual bias is largely systematic across conditions. Because our inferences rely on relative comparisons among methods rather than absolute percentages, this limitation does not change our main conclusions.

**Reviewer #2:**

*The manuscript titled “Evaluation of RNA extraction and rRNA depletion protocols for RNA-Seq in eleven edible seaweed species from brown, red, and green algae (Manuscript No. PONE-D-25-34674)” provides a valuable cross-species methodological comparison. At the same time, I feel there is no need of this paper altogether, as literature on RNA extraction for various seaweeds are already available.*

*Coming to the article, the study is well-structured and offers practical guidance for researchers working on non-model algal species (though there are hundreds of seaweeds species exists, and studies have been done only on 11 including 6 brown algae). However, several aspects require further clarification and refinement to ensure reproducibility, transparency, and broader applicability. Please address the following comments.*

We thank the reviewer for their constructive evaluation and for highlighting the strengths of our work. We appreciate the concern regarding the paper’s necessity. Although multiple protocols for RNA isolation from seaweeds have been published (refs. 10, 12–18), we encountered substantial challenges across the 11 species examined, and selecting an optimal method was not straightforward. Accordingly, our goal was to provide a cross-species comparison that includes a representative selection of methods reported in previous studies. Furthermore, to our knowledge, no commercial rRNA-depletion tools are specifically designed for macroalgae; a systematic evaluation of general-purpose ribodepletion protocols in this context has been lacking. The rationale for our study has now been expanded (L63-74), thereby also addressing the concern raised by reviewer #3.

***1. The Introduction section contains results (lines 77–84) that should be removed to maintain focus on the background and study rationale. Point on RNA virus in the first para of introduction is a misfit. If you want to emphasize on RNA viruses, start with a fresh paragraph.***

We thank the reviewer for this helpful comment. In some research areas with which we are more familiar, it is common practice to summarize key results and conclusions at the end of the introduction. However, we now recognize that in this field such information is not typically included in the introduction. We have therefore removed the results from lines 77–84. In addition, we judged that elaborating on RNA viruses in a separate paragraph would not align with the main focus of this paper and thus removed the sentence.

***2. The study evaluates RNA isolation and rRNA depletion protocols across 11 seaweed species; however, the sampling is skewed toward brown algae (6 species) compared to red algae (4 species) and green algae (1 species). This taxonomic imbalance could bias conclusions toward protocols that perform better for brown algae. Please discuss this limitation and whether the observed trends are likely to hold across a more balanced species representation.***

We agree that our sampling is weighted toward brown algae (6 brown, 4 red, 1 green) and that this affects how broadly results can be generalized. This limitation is now explicitly acknowledged, emphasizing that although results observed within a particular colour group may hold for the taxa examined, they cannot necessarily be generalized to all species within that group (L283-289).

***3******. To strengthen the evaluation of RNA isolation and rRNA depletion protocols, we recommend applying a more rigorous statistical framework.***

We agree with the reviewer, as we also preferred to apply a rigorous statistical frame work, but after consulting with our biostatistician during the course of this project, we came to the conclusion that this is not readily implementable for the following reasons:

- For our main QC readouts (RNA integrity, yield, OD260/230, and OD260/280) there are, to our knowledge, no *de facto* standard statistical methods available.
- We have three technical replicates per condition, and they’re noisy. This matches our experience: “easy” samples give very consistent QC, while difficult tissues vary more even when treated the same way. Assessing outlier-behaviour of samples is in this context based on laboratory experience. In order to give maximum transparency to the readers, we have therefore provided all individual QC values in S5 and S6 Table.
- For the final advice for which protocol to use on which species, we have given different priorities to different QC metrics (as described in L189-194). This is difficult to take into account in a formal statistical test and has limited added value. Readers can interpret the results by assigning their own relative importance to the various QC metrics and select the method that best aligns with their specific goals. For example, if large quantities of material are required for multiple downstream assays, yield may be prioritized over integrity or purity. A similar study by Jensen *et al.* ([15] in our paper) used the same metrics and also did not apply statistical tests.

Our goal was practical: identify the protocol that is most likely to work across challenging samples, and give the reader insight we use descriptive statistics rather than inferential.

***4. The discussion could be strengthened by hypothesizing why certain species (especially some red algae) yield poor RNA with specific methods, referencing known polysaccharide or polyphenol interference.***

Our original aim was to keep the paper focused on the practical selection of working protocols; however, we now recognize that a more extensive discussion of the results would strengthen the manuscript. We have therefore added a biochemistry-focused paragraph to the results/discussion that more extensively discusses our results (L157-179).

***5. In result, starting material used is written as 150 microgram and in method its 150 mg. Which is correct?***

Thank you for catching this error: 'microgram' should have been 'milligram' (L113).

***6. The manuscript reports RIN values and absorbance ratios for RNA quality assessment but does not discuss whether these meet accepted RNA-seq input quality thresholds. Please address how the measured values align with recommended standards, and if any deviations occurred, discuss potential implications for downstream RNA-seq performance.***

To our knowledge, there is no single official standard for RNA QC metrics accepted for RNA-seq. Most recommendations for minimum RNA yield and integrity are provided in the manuals of library preparation kits supplied by manufacturers. OD260/280 and 260/230 ratios serve as more general indicators of RNA purity used for different applications. Several studies suggest broadly similar QC requirements that apply to gene expression analyses in general. We have included a brief discussion of this point and cited the relevant references to inform readers that guidelines on this matter exist (L181-186).

***7. While the manuscript provides a robust technical comparison of RNA isolation and rRNA depletion protocols, it would benefit from including practical considerations such as approximate cost per sample, processing time, and ease of use for each method. This information would make the conclusions more actionable, particularly for laboratories with limited budgets or resources.***

Thank you for this suggestion. We agree that cost information is valuable for readers. We have added a paragraph discussing the costs of the different protocols and kits, along with a rough comparison of their labour requirements (L263-273), to help readers quickly determine which approaches are generally more economical.

**Reviewer #3**

*Overall, the manuscript addresses an important methodological framework in algal transcriptomics by systematically evaluating RNA extraction and rRNA depletion protocols across a diverse set of seaweed species. The manuscript is generally well structured and the experimental design appears rigorous. However, there are areas where clarifications are needed before the manuscript can be accepted.*

We thank the reviewer for carefully reading our manuscript and for offering valuable suggestions and corrections, which we believe have substantially enhanced the manuscript.

***Introduction***

*Pg. 2, Line No.30-32: The authors stated that “advancing our understanding of seaweed biology, including its response to environmental stressors and pathogens, is essential for enhancing cultivation practices.” Please clarify which stressors (temperature, salinity, pollutants) have already been studied in seaweeds, and which remain underexplored?*

We acknowledge that, in hindsight, the introductory paragraph did not adequately represent the current state of research across the different areas. It was not our intention to suggest that little research has been done on seaweed physiology and environmental stressors; on the contrary, this field is well established. Rather, our point was that studies employing genomics and transcriptomics approaches (especially to investigate pathogen interactions) remain relatively limited. We have therefore revised the text to remove any such unintended implications and to provide a more accurate and concise overview of some relevant research fields (L30-35).

*Pg. 2, Line No.39-44: The authors have mentioned “RNA-seq limitations in capturing non-polyadenylated RNAs. Since many algal viruses and prokaryotic RNAs lack polyA tails, could the authors provide evidence from prior algal/seaweed transcriptome studies where viral transcripts were missed due to polyA-based methods?*

Thank you. To our knowledge, direct demonstrations in literature of “missed entirely” cases in seaweeds are not available. We have revised the text to avoid implying this and now reference algal metatranscriptome studies showing that poly(A)-selected libraries recover substantially fewer viral reads and viral contigs compared to ribodepleted total RNA libraries (L42-48). Briefly, in a brown-tide algal bloom, reads mapping to the *Aureococcus anophagefferens* virus were ~2 orders of magnitude lower with poly(A) selection than with ribodepletion; the authors therefore recommend rRNA-reduced approaches for community-level virus analyses (Gann et al.). In addition, recent algal virus discovery studies have employed total-RNA/rRNA-depleted metatranscriptomes rather than poly(A) selection, consistent with the need to detect non-polyadenylated viral transcripts (Charon et al.).

*Pg. 2, Line No.45–61: Have there been any comparative evaluations of these methods specifically in seaweeds before? If not, please make clear that this study is addressing the first comparison report.*

We identified seven studies that focused on optimizing RNA isolation for individual seaweed species or color groups. One additional study evaluated total RNA extraction across multiple seaweed taxa, but it compared only two protocols (CTAB- and SDS-based). From the eight referenced studies, we selected a representative set of protocols, excluding those with only minor variations from methods already included. In addition, to our knowledge, no systematic comparisons of ribodepletion strategies in seaweeds have been published to date, and no commercial ribodepletion kits are currently designed specifically for algal samples. We updated the introduction to outline our protocol selection rationale and to underscore that a more extensive selection of protocols coupled to downstream ribodepletion makes this study unique (L64-69).

***Methods***

*Pg. 11, Line No. 245–251: The authors mentioned flash-freezing in liquid nitrogen and storage at -80 °C. Since long-term frozen storage can sometimes affect RNA quality in high-polysaccharide tissues, was RNA integrity assessed immediately after storage to ensure no degradation during sample handling?*

We share the reviewer’s observation that extended storage can, in some cases, affect RNA quality. We harvested fresh material, transported at 4 °C to the laboratory and immediately frozen at −80 °C upon arrival. All RNA isolations were completed within three months of storage. The triplicate RNA isolations were performed across this storage period, and no clear correlation was observed between RNA integrity and storage duration (S5 Table). The maximum storage duration has now been specified in the Materials and Methods (L308-310).

*In CTAB methods, do authors measured the carryover of polysaccharides or polyphenols (e.g., by A230 contamination ratios or carbohydrate assays) to confirm the effectiveness of CTAB-based removal?*

We did not carry out specific assays to quantify polysaccharide or polyphenol content in the isolates. Instead, we monitored absorbance at 230 nm, expressed as OD230/260 ratios, which are typically depressed in the presence of polysaccharides. (Poly)phenols, by contrast, can lower either the OD230/260 or OD260/280 ratios. We therefore used both ratios as indicators of overall RNA purity, while recognizing that they are not definitive markers of particular contaminants.

*In LogSpin method, any RNase inhibitor was added during extraction, since guanidine-based buffers can still leave trace RNase activity in seaweed tissues?*

No RNase inhibitors were added, as the procedure was followed exactly as described in the original publication. It is generally accepted that lysis in 8 M guanidinium fully denatures RNases, and would likewise inactivate protein-based RNase inhibitors. However, we agree that once the chaotropic salt is removed during subsequent steps, endogenous RNases may refold and regain activity, leading to RNA degradation in the final preparation or downstream reactions. In our study, chaotropic lysis/column-based extractions consistently underperformed for brown seaweeds, but this is more plausibly explained by their high polysaccharide and polyphenol content rather than RNase activity alone. We note that if any residual RNase activity affected RNA quality in the final preparations, this would be inherent to the method itself and therefore forms part of our evaluation.

*Pg. 15, Line No.350–353, the cutoff of 90% coverage relative to the highest coverage contig was used. How was this threshold determined?*

We acknowledge that this point was missing from the text. The choice of a 90% coverage cutoff was not based on a systematic optimization; instead, at this threshold, contigs showing homology to all major nuclear and mitochondrial rRNA species were consistently captured in the rRNA reference proxies. We have now included a brief explanation (L212-214).

***Results and Discussion***

*Page 5, Line No.108–110: CTAB-based methods generally yielded higher RNA quantities but did not consistently align with acceptable RNA integrity. What underlying molecular or biochemical factors could cause this discrepancy? Please discuss.*

There are various possible explanations for this observation. Bulk precipitation using alcohol in CTAB protocols is non-selective, recovering intact RNA, fragments, DNA, and contaminants. In contrast, silica columns are more selective but often clog or under-bind with polysaccharide-rich macroalgae lysates, reducing yield. CTAB complexes polysaccharides, tolerates more tissue/lysate, and avoids strict capacity limits, thereby increasing recovery. However, CTAB involves more handling before precipitation, allowing RNase activity and mechanical/chemical damage that can lower RNA integrity, especially for the longer rRNAs that are used as a proxy for total RNA integrity. We have added a brief discussion including these arguments to the manuscript (L114-119).

*Page 5, Line No 111–113: Spin-column methods performed better in red seaweeds. What specific traits of red seaweeds influenced their compatibility with spin-column methods compared to CTAB-based ones?*

A more detailed discussion of this observation, as well as other issues highlighted by reviewer #2, has been added in a separate paragraph. (L156-178).

*Page 6, Line No 135-141: Methods involving phenol/chaotropic salts showed suboptimal purity in brown seaweeds. how was this validated? as residual contaminants directly affect downstream ribodepletion or sequencing efficiency.*

As noted earlier, the substantially lower purity of column-based methods in brown seaweeds was determined solely by spectroscopic OD260/230 and OD260/280 ratios. According to widely accepted RNA-seq standards (see also reviewer #2, comment 6), RNA preparations with such poor OD ratios are not recommended. Consequently, these low-purity samples were not used for RNA library preparation; instead, only the highest-quality samples and methods were selected, given the considerable cost of downstream protocols.

*Overall, the authors need to provide a more detailed discussion of the results obtained.*

Taken together, all reviewer comments highlighting the need for a more detailed discussion of the results have been incorporated into the manuscript, which we believe enhances the value of our findings for readers.

**Reviewer #4**

***Abstract***

*The genus names of Seaweeds has to be mentioned for more clarity along with the phylum name. (Line nos 17 & 18)*

We appreciate this comment and have included the corresponding phyla at the first mention of the color groups in the abstract and introduction.

***Introduction***

*In Line no. 31 Include as “Environmental Stress factors….”*

The corresponding paragraph was revised to address another reviewer’s comment, and we now use the term ‘environmental stress responses.

*Line nos 78 – 84 can be removed from Introduction part and may be included in Results and discussion column.*

We have removed these results from the Introduction.

***Results and Discussion***

*Nano drop quantification may be included in the methodology for the justification of purity of RNA.*

We have included this information in lines 376-378.

*Line no 144 Title has to be precise and meaningful.*

We have renamed this section to “Recommended RNA extraction workflows”.

*Kindly justify the novelty of the work.*

We appreciate this suggestion and have included a short justification in lines 64-69.

*Fig 1 & 2 Standard errors may be provided in the figures.*

Unfortunately, unlike the RNA extractions, which were performed in triplicate, the ribodepletion and sequencing were carried out without replication due to the high costs involved. The study was already very costly for the financial resources we have available, as the large number of sample–ribodepletion combinations resulted in a total of 43 sequencing libraries.
