# Supplementary material for: Evaluation of RNA extraction and rRNA depletion protocols for RNA-Seq in eleven edible seaweed species from brown, red, and green algae
Source: PLoS One. 2026 Jan 2;21(1):e0339896. doi: 10.1371/journal.pone.0339896 (PMC12758695; doi:10.1371/journal.pone.0339896)
Supplement: Attachment — Submitted filename: Comments RNAseqRevised.docx [file pone.0339896.s011.docx]

Recommendation

The manuscript may be accepted as the authors given complete reply for the reviewers comments and modified the manuscript as per the comments. The manuscript can be accepted in this present manner.
